# Bing–Neel syndrome, a rare manifestation of WM; a case report and review of literature

**DOI:** 10.1002/ccr3.9034

**Published:** 2024-06-04

**Authors:** Hamid Rezvani, Sina Salari, Hamed Borhani, Maedeh Mataji, Hamed Azhdari Tehrani

**Affiliations:** ^1^ Department of Hematology‐Medical Oncology Shahid Beheshti University of Medical Sciences Tehran Iran

**Keywords:** Bing–Neel syndrome, low‐grade lymphoma, Waldenström macroglobulinemia

## Abstract

Bing–Neel syndrome (BNS) is a rare manifestation in individuals suffering from Waldenström macroglobulinemia (WM). Neurological signs and symptoms in this syndrome are almost difficult to be differentiated from other common neurological manifestations of hyper‐viscosity or Waldenström‐associated polyneuropathy. In this paper, we report a new case of WM with concurrent BNS, then review the clinical picture and treatment of this syndrome.

## 1 INTRODUCTION

Bing–Neel Syndrome (BNS) is a rare manifestation of Waldenström macroglobulinemia (WM) that occurs approximately in 1% of patients with WM. Manifestations can occur simultaneously or subsequently after primary WM treatment.^1^ The first case was diagnosed almost 8 years before the introduction of WM in 1936. Patients with this syndrome can be referred with neurologic signs and symptoms related to the loco‐regional brain involvement or can be asymptomatic despite the involvement of brain parenchyma or cerebrospinal fluid.^2^ In several retrospective series or case reports the interval between asymptomatic involvement to obvious signs or symptoms lasting almost several months. BNS symptoms may develop in patients receiving cytotoxic chemotherapy for WM treatment.^3^ Here, in this report, we present a middle‐aged man with WM and concurrent BNS. Also, we review the literature at the end.

## 2 CASE HISTORY

A 56‐year‐old man was referred to our emergency department about 1 month ago with severe headache, vertigo, nausea and vomiting. In the initial evaluation, the body temperature was 37.4°C, the blood pressure was 130/80 mmHg, and the respiratory rate was 18/min with a heart rate of 90 per minute. During the physical exam, there were no abnormalities, but the examination was not done completely due to poor cooperation. The Laboratory examination showed mild leukocytosis (14.5 × 10^9^/L) along with normocytic normochromic anemia (Hemoglobin 8.4 g/dL and MCV 93 fL and MCH 32 pg) and thrombocytopenia (89 × 10^9^/L). The renal function test showed some abnormality (Cr 1.9 mg/dL and BUN 85 mg/dL) and liver function test also revealed rising in Alanine aminotransferase (89 IU/L) and aspartate aminotransferase (122 IU/L). There was no electrolyte abnormality (Table 1).

**TABLE 1:** Important laboratory parameters.

Laboratory parameters	Value
WBC	14.5 × 10^9^/L
Hb	8.4 g/dL
PLT	89 × 10^9^/L
Cr	1.9 mg/dL
AST	89 IU/L
ALT	122 IU/L
ALP	548 U/L
Bilirubin Total/Direct	1.4/0.3 mg/dL
Electrolytes	Normal
Lymphoma Stage	IV

Abbreviations: ALP, Alkaline Phosphatase; ALT, Alanine Aminotransferase; AST, Aspartate Aminotransferase; Cr, Creatinine; Hb, Hemoglobin; PLT, Platelets; WBC, White Blood Cells.

## 3 DIAGNOSIS AND TREATMENT

**FIGURE 1:**
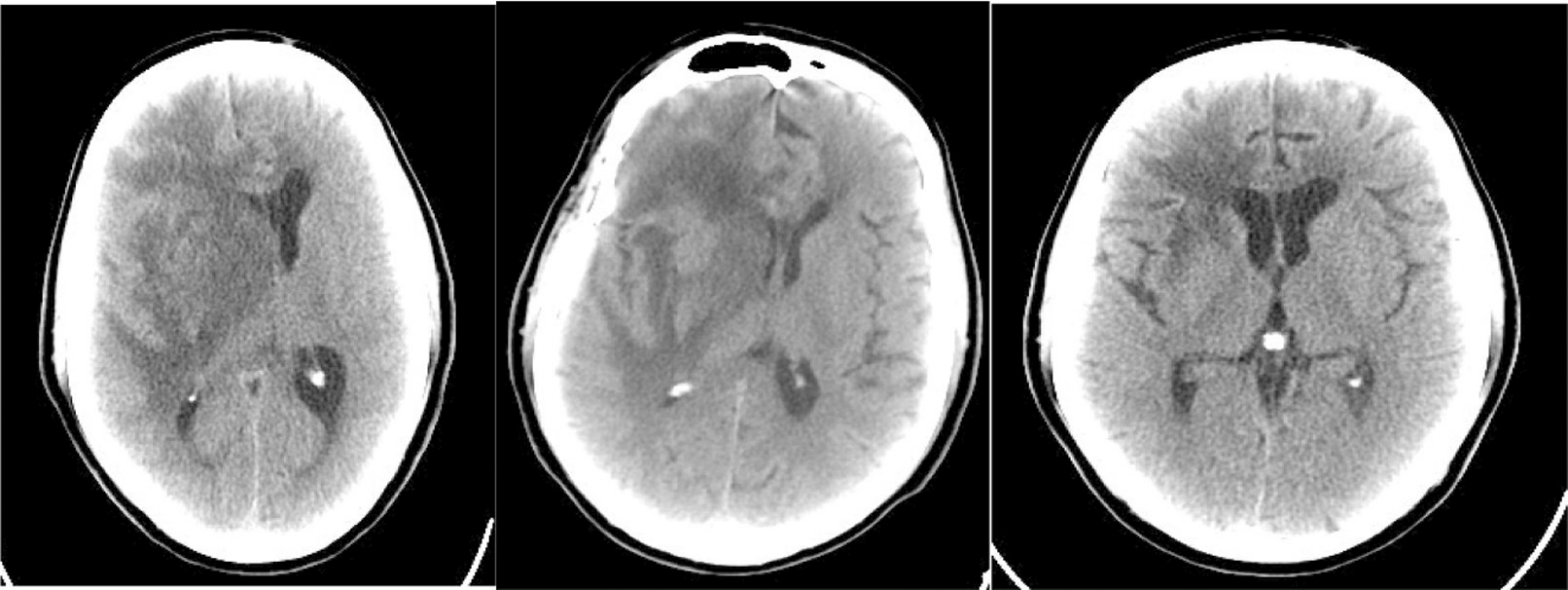
Brain CT involvement at the time of the diagnosis and response to the treatment. Left: Diffuse Fronto‐temporal vasogenic edema at presentation. Middle: After two sessions of plasma exchange in week one. Right: One week after first course of systemic chemotherapy.

**FIGURE 2:**
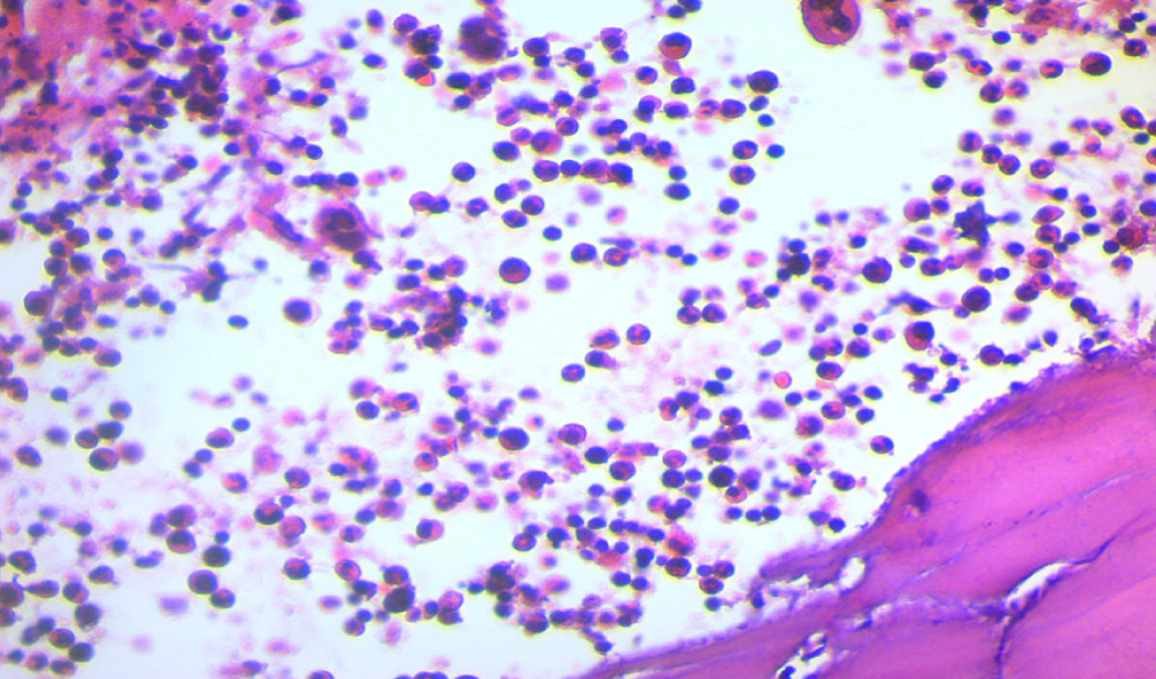
Bone marrow biopsy.

**FIGURE 3:**
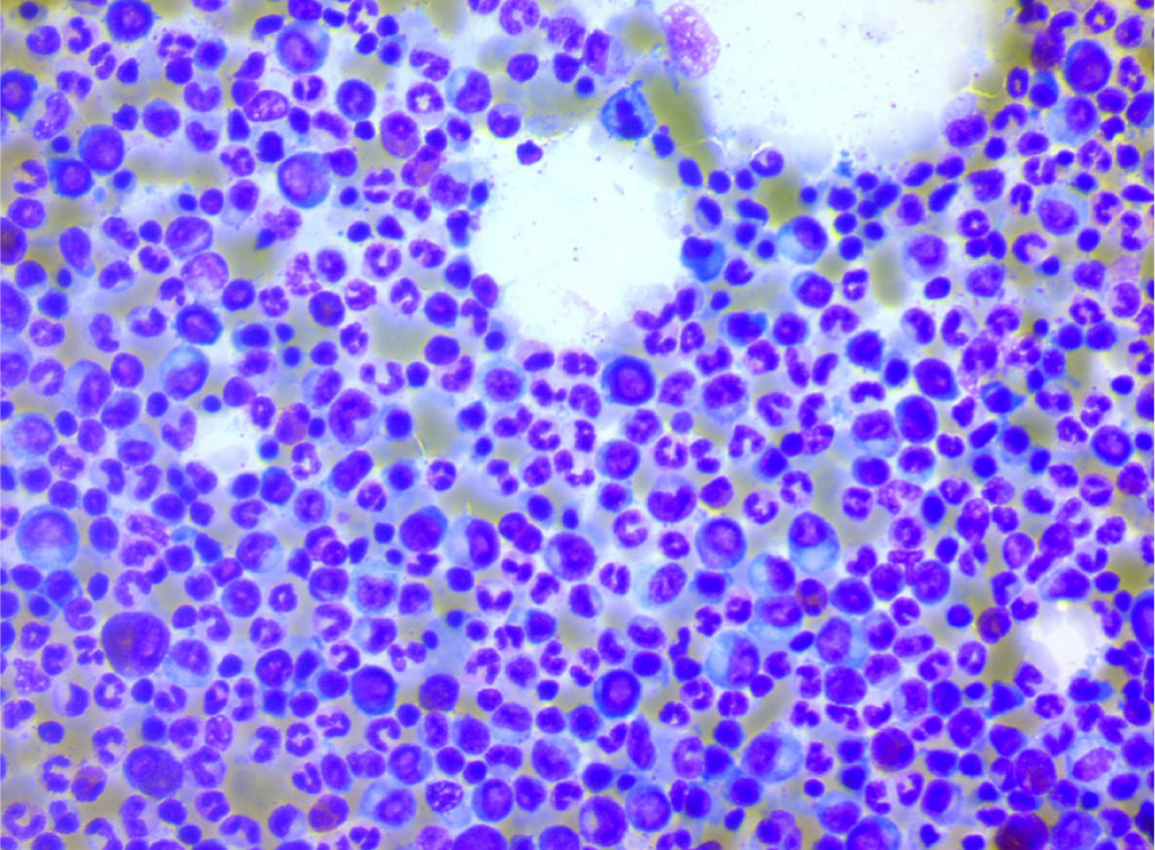
Bone marrow aspiration.

Brain CT scan without contrast showed intra axial mass in right fronto‐temporal lobes with massive edema and midline shift (Figure 1). Serum protein electrophoresis showed a sharp peak in gamma, and immunofixation revealed an IgM monoclonal origin (12.9 g/dL). Due to the monoclonality of immunoglobulins, bone marrow aspiration and biopsy were done. The patient's bone marrow showed about 60% lymphoplasma cells that were positive for CD20 (+) and CD138 (+) (Figures 2 and 3). No osteolytic bone lesions were found in association with multiple myeloma. Calcium (9.8 mg/dL) and other electrolytes were normal. Regarding permission from the neurologist to evaluate CSF through lumbar puncture, CSF analysis was performed. Microscopic examination showed numerous plasma cells, and flow cytometry confirmed it. MYD88 mutation status analysis was not present in our laboratory. Regarding the high concentration of IgM, we started plasma exchange (40 cc/kg) for two sessions. The level of consciousness was slightly improved. In addition to the plasma exchange, chemotherapy with high‐dose methotrexate (3 g/m2) and Bendamustine (90 mg/m2 d1–d2) was initiated. To avoid cytokine storm, Rituximab was not administered in the first cycle. After 2 weeks, the patient's condition had a significant improvement. After a period of 2 weeks, serum IgM was checked again and the result was 4 g/L. Rituximab (375 mg/m2 d1) added to Bendamustine in the second cycle. The patient received Bendamustine and Rituximab every 3 weeks alternating with high dose of methotrexate (3 g/m2) every 2 weeks. Now, the patient is currently treated with this combined regimen. The following brain CT scan demonstrated a response to treatment and a decrease in vasogenic edema (Figure 1).

## 4 DISCUSSION

Bing–Neel syndrome (BNS) is an unusual presentation in WM, and it can present in two different types in patients; parenchymal involvement with tumor‐like lesions that can present with loco‐regional manifestations or infiltrating lesions that can produce vasogenic edema, which present with elevated intracranial pressure (ICP) and some non‐specific signs and symptoms. Another presentation is leptomeningeal involvement.^3^ The initial approach is to obtain brain and spine magnetic resonance imaging (MRI) with intravenous Gadolinium (GAD), especially in T1 to exclude the other possible diagnoses and also cerebrospinal fluid (CSF) analysis if possible. The key element for diagnosis of BNS is the presence of malignant lymphoplasmacytic cells (LPCs) in the CSF cytology or brain tissue biopsy; however, tissue biopsy is not routinely necessary.^4^, ^5^ The typical diagnostic markers, which are the same as the WM diagnostic markers that are CD19, CD20, CD22, CD79a, and CD79b for lymphocytes and also positive in staining for CD138 and IgM for plasma cells.^6^ Similar to WM, molecular studies with polymerase chain reaction (PCR) reveal that these cells can express MYD88 in more than 90% of patients and also have rearrangements in immunoglobulin heavy chain (IGH).^7^ Imaging manifestations of this syndrome were mentioned above; however, normal MRI cannot exclude the diagnosis of BNS. Another point to consider is MYD88 mutation, and lymphocytic flow‐cytometric markers can become positive in other lymphoproliferative disorders, like marginal zone lymphoma (MZL) or chronic lymphocytic leukemia (CLL).^8^, ^9^ The diagnosis of BNS could be challengeable especially with the exclusion of other possible diagnoses. Compared to patients with WM, Data on CXCR4 gene mutation in these patients is limited.^10^ There are some differential diagnoses with BNS such as hyper‐viscosity syndrome, which is associated with high levels of IgM (more than 4 g/dL) and also nonspecific central nervous system manifestations. The second one is polyneuropathy, which can be related to IgM and is usually symmetrical ascending progressive sensory polyneuropathy. However, in BNS, the concentration of IgM in CSF is usually less than 1 g/dL. IgM‐associated neuropathy can be related to anti‐myelin‐associated glycoprotein (MAG) antibodies.^11^, ^12^

Treatment is necessary in symptomatic patients; however, it is unclear whether treatment is necessary in asymptomatic patients with lymphoplasmocytic cells in CSF or central nervous system.^3^, ^13^ It is unclear if we should perform brain MRI or lumbar puncture for the patients with WM to exclude the CNS involvement. Up to now, the current management of WM does not recommend to evaluate CNS. In the literature, the evaluation was based on the trials and it would be different in the real world clinical practice to evaluate which patient. However, due to little population of these patients with this clinical manifestation, conclusion of whether these patients need specific treatment or not is too difficult. So the asymptomatic patients cannot be really and easily diagnosed and excluded of treatment.

The goal of therapy in WM as an indolent lymphoma is to prolong progression‐free survival (PFS), so BNS can be treated like WM and complete eradication is not feasible or mandatory.^14^ The treatment strategy for BNS is not clearly determined; however, based on previous experience in the treatment of lymphomatous involvement of the central nervous system, we use the same treatment approaches such as high‐dose methotrexate or Cytarabine, because of potential CSF penetration.^15^ Alkylating agents such as Bendamustine or nucleoside analogous such as Fludarabine or Cladirabine, have been found to be active, but they have negative impact on marrow cells.^3^ New reports on the efficacy of Ibrutinib in the primary management of BNS have been published, and this agent could prolong event‐free survival (EFS) and also overall survival (OS). Castillo et al.^16^ reported 34 patients with BNS that were treated with high‐dose methotrexate in 41% and high dose of Cytarabine in 16%, and 19% were treated with intrathecal methotrexate. Objective response rate was 66% and 3‐year‐overall survival was 59%. Laurence Simon et al. reported a case series of 44 patients with BNS who were treated with various chemotherapeutic regimen such as high‐dose methotrexate or Cytarabine, CHOP (Cyclophosphamide, Doxorubicin, Vincristine, Prednisolone), Fludarabine‐based regimen in combination with rituximab, etc. 91% of patients received high‐dose methotrexate or Cytarabine. 73% of patients also received intrathecal chemotherapy. The overall response rate was 70% (with one patient experiencing complete response (CR)), and the five‐year overall survival was 71%.^1^ Again, Castillo et al. reported 28 patients with BNS that were treated with Ibrutinib (46% of patients received 560 mg and 54% received 420 mg daily). Three months of therapy resulted in improvement of symptoms, radiology, and CSF cytological clearance by 85%, 60%, and 58%, respectively. Two years of overall survival and event‐free survival were 81% and 80%, respectively.^5^ A new report from a Polish lymphoma research group evaluated 11 patients with BNS who were treated with high‐dose methotrexate or Cytarabine (mostly), ibrutinib and other agents such as Bendamustine or Rituximab. Four patients had a partial response, and four patients had a complete response or uncertain complete response. The median survival was 28 months, and the 24‐month overall survival was 60%. Three years of overall survival was 47%.^15^

In this paper, we reported a middle‐aged man with WM and CNS involvement that was responded well to high‐dose methotrexate and Bendamustine plus Rituximab yet. In this case, the serum IgM was higher than expected value in case of BNS; however, due to brain parenchymal involvement and also massive vasogenic edema, we decided to treat this case as same as BNS. The patient has responded well up to now, and the treatment was scheduled for six cycles of Bendamustine plus rituximab and high‐dose methotrexate. Evaluation would be done after three cycles of chemotherapy.

## 5 CONCLUSION

In conclusion, BNS, as an unusual involvement that is seen in WM, has several challenges in diagnosis and treatment. Studies with larger samples are necessary to clarify these issues.

## AUTHOR CONTRIBUTIONS

**Hamid Rezvani:** Conceptualization; data curation. **Sina Salari:** Writing – review and editing. **Hamed Borhani:** Investigation. **Maedeh Mataji:** Writing – original draft. **Hamed Azhdari Tehrani:** Writing – original draft.

## FUNDING INFORMATION

The research received no funding grant.

## CONFLICT OF INTEREST STATEMENT

The authors declare that they have no conflict of interest.

## ETHICAL APPROVAL

All procedures performed in this study were in accordance of ethical standards of Helsinki declaration.

## CONSENT

Written informed consent was obtained from the patient to publish this report in accordance with the journal's patient consent policy.

## Data Availability

Data sharing is not applicable to this case report type article as no new data were created or analyzed in this study.
