# Correction to “Bing–Neel syndrome, a rare manifestation of WM: A case report and review of literature”

**DOI:** 10.1002/ccr3.9109

**Published:** 2024-07-12

Rezvani, H., Salari, S., Borhany, H., Mataji, M., & Azhdari Tehrani, H. (2024). Bing–Neel syndrome, a rare manifestation of WM: A case report and review of literature. *Clinical Case Reports*, *12*(6). https://doi.org/10.1002/ccr3.9034

In the field of author's name, the third author's family name typed in a wrong dictation.

The correct name is Hamed “Borhany” which ends to “Y”.

Borhani which ends to “i” is incorrect, and correction is necessary.

We apologize for this error.

